# Euchinin—A New Preparation of Quinin

**Published:** 1897-04

**Authors:** 


### Euchinin—A New Preparation of Quinin.

Dr. O. Van Noorden, in *Centralblatt fur innere Medicin*, November 28th, says that a preparation of quinin, called euchinin, possesses the curative properties of quinin without having its bitter taste or producing a disturbed appetite, nausea, noises in the ears, heaviness, etc. It is formed by the action of ethylchlorocarbonate on quinin. Euchinin crystalizes in needles, and is readily soluble in alcohol, ether or chloroform, but it is with difficulty dissolved by water. The chlorid of euchinin is easily soluble in water, the sulphate is less soluble, and the tannate least so. The base unchinin is tasteless unless it remains on the tongue for a long time, when it is slightly bitter. If given in sherry, milk or cocoa, it produces no unpleasant taste. Healthy people can stand 1 gram. and usually 2 grams, without unpleasant effects. Once 2 grams caused a sensation of heaviness in the head. No unpleasant results were noted in patients, and no complaint was made during the prolonged use of the larger doses of 1 to 2 grams in the day. In the absence of cases of malaria, the author has tried euchinin in fifteen cases of whooping-cough, fourteen cases of hectic fever in phthisis, five cases of sepsis of various origin, in pneumonia, typhoid fever, and also in several cases of neuralgia. One gram of quinin has the same action in whooping-cough and in febrile states as 1½ to 2 grams euchinin. Beneficial and rapid effects were noted in twelve cases of whooping-cough. The temperature had a lower range in the fever cases after a few days. The results were especially good in one case of supraorbital neuralgia. A dose of 0.6 of quinin or 1 dram of euchinin gave relief when all other nervine tonics failed. This result was seen in eight different attacks. Euchinin is best given in tabloid form. In children it may be given in milk, soup or cocoa. The tannate is much to be recommended. The author thinks that euchinin certainly deserves a further trial, and that it will prove an addition to our resources.

A tumor in the mouth was about to be extirpated when it opened spontaneously and discharged a calculus, which Lavoine assumes had originated in Wharton's duct.—*Nord Med.*

